# Success and survival conference: a novel idea to resonate an underutilized concept in medical education

**DOI:** 10.5116/ijme.5969.c8ec

**Published:** 2017-07-20

**Authors:** Mohammed Elhassan

**Affiliations:** 1 Department of Internal Medicine, Division of Hospital Medicine, UCSF/Fresno Center for Medical Education and Research, USA

**Keywords:** Success and survival, conference, educational activities, appreciative inquiry, learning from excellence

## Introduction

Since the publication of the Flexner Report on medical education and the creation of the Hospital Standardization Program by the American College of Surgeons in the early 1900s, morbidity and mortality (M&M) conference started to evolve gradually into medical education. Nowadays, it is a well-recognized educational activity which is usually taught periodically to improve medical education in both surgical and medical residency training programs in the United States and worldwide. The Accreditation Council for Graduate Medical Education (ACGME), the organization responsible for the accreditation of postgraduate medical training programs in the United States, made it a requirement in 1983 for all training programs to conduct M&M rounds, or similar quality improvement and patient safety conferences, on regular basis in order to meet accreditation standards.

The process of reflecting upon medical errors and deficiencies, with all the anxiety and stress that can be associated with it, is of upmost importance for professional development and maturation. Equally important, though, is the concept of appreciative inquiry (AI) and “learning from excellence”. One definition of AI is “the cooperative search for the best in people, their organizations, and the world around them. It involves systematic discover of what gives a system ‘life’ when it is most effective and capable in economic, ecological, and human terms.”^1^ In other words, the AI approach, in contrast to the problem-solving approach, is based on highlighting the strengths (rather than the weaknesses) of institutions and the right actions (rather than the wrong actions) that were done to reach the specified goals. The M&M conference is an example of a practical application of the problem-based approach which has become widely accepted as an educational method.

This concept of AI has long been used and studied in business and organizational development, but it seems that it is not widely utilized by the medical education community.^2^ Thinking in that direction, medical educators should not only teach learners how to ponder over their mistakes and deficiencies (which the M&M rounds serve), but should also emphasize to them that there is also a lot to learn from excellence, hard work, and walking the extra mile for patients to create a successful patient management story. Medical educators probably do this intermittently during clinical rounds, in one-to-one meetings, during feedback sessions with their learners, or during any other encounters where patient care is being discussed. Nevertheless, the same way we have structured M&M conferences on a regular basis to pause and learn from our mistakes, we should adopt and build a dedicated structure that equally highlights the hard work of learners and the excellence of endeavor that they exhibit at times. That hard work can be displayed by them in many different ways, such as extensively examining literature related to patients’ medical problems to solve a medical puzzle or to conduct the best evidence-based treatment plan; making phone calls or sending emails to different providers who took care of their patients in the past to gather necessary data that would have not been retrieved otherwise; or making extra effort to obtain an important test, device, or therapy for patients who need them but are not able to afford them. Such dedicated practical application which utilizes the AI concept is expected to have a positive influence on the learners’ educational and training experience. I suggest to name such a meeting or conference the “the Success and Survival”, or the “S&S” conference, and in this paper, I would like to introduce this idea to medical educators and learners globally.

### S&S Conference: the “how”

The proposed S&S conference can be similar in built and structure to the well-known M&M conference, with the major difference being the goal. M&M conference intends to focus on “what went wrong and how can we prevent this from happening again”, while and S&S conference targets good practice and behavior and answers the questions of “what went right and how can we maintain this practice, disseminate it and reinforce it). Also similar to the M&M, the S&S conference should be standardized when conducted. That will help medical educators study its effect and impact after implementation with the intention to improve the conference and make it more informative and productive. Faculty should be involved in the selection of cases to be presented in the conference to make sure that the educational value of the case is valid and genuine. At times, the educator will be the one who should suggest to the team to prepare a case which is thought to fit the purpose of the conference, especially if the educator believes that the team might be “too shy” or “too humble” to talk about their hard work and the good outcome they achieved in treating a patient or dealing with a challenging clinical situation. A general matrix or guidelines should be followed during the presentation. These guidelines should be individualized for each program and should be agreed upon by the program’s medical educators. They should also highlight and embrace the goals of the conference. I also suggest that, like the M&M rounds, the six core competencies adopted by the ACGME (patient care; medical knowledge; practice-based learning and improvement; interpersonal and communication skills; professionalism; and systems-based practice) be inseminated into the matrix of the conference to help learners understand what these competencies mean and how they can achieve these expected milestones throughout their training years.

Training programs outside the United States can use their agenda which followed the guidelines posted by their own regulatory bodies. Emphasis should be stressed more upon the effort made by the whole team but without ignoring the individual work and achievement. Keeping that balance might be challenging but this goal can be highlighted by having more than one resident or having the whole team members present the case during the conference. Moreover, nurses and other ancillary staff members should also be invited to participate in the conference if they were involved in the case or the situation being discussed. “Thank you” letters received by residents and the medical staff which is sent by some patients or their families, can also be portrayed and read in S&S rounds to show appreciation and gratitude to the team who cared for the patient.

### S&S Conference: the “why”

Educational activities such as S&S rounds can have many potential benefits for medical students and residents. First, we can potentially learn a lot from our peers, and we learn by example.  “Peer pressure,” when it comes to positive values and attitudes, is a healthy phenomenon. Relating peers’ praised practice to “success” and “survival” will form a foundation for other medical residents to try to adopt the same practice and will encourage them to follow the steps of their fellow residents in what they did to come up with that successful patient story. Second, S&S conferences can serve as a mesh to help residents build their confidence in their clinical abilities. It can assist them to realize that they are not “just residents” and can indeed make a difference in the outcome of their patients in all different clinical settings: in-patient, out-patient, or with specialized consultants. Third, the learners will be assured that their residency program is not only running after their mistakes (to help them learn from those mistakes) but also is “running after their strengths” and it does appreciate it when they push themselves further to deliver better care. Such appreciation when presented professionally to others, with preservation of residents’ modesty and unpretentiousness, can create a stronger bond between them and their program. Fourth, the importance of team work and communication will be emphasized. Almost all successful patient stories are the yield of a hard effort of more than one person working together and helping each other in delivering better medical care.

## Conclusions

Medical educators should start building specific educational structures that embrace notions such as “appreciative inquiry” and “learning from excellence” more often than currently held in teaching agenda and training program curricula. The S&S conference, in line with the popular and well-known M&M conference, is one novel practical idea that can be inaugurated to add to the constructive and profitable medical educational experience during residency training of internal medicine or any other specialities.

Introducing the initiative to the leadership in the internal medicine residency training program here in Fresno, CA has yielded positive reception, and we are in the process of implementing it during the “educational half-days” and on Fridays’ noon lectures, where our usual M&M rounds take place. Post-implementation surveys and re-evaluations can help reveal defects and deficiencies that should be analyzed and repaired with the goal of maximizing educational benefit and attaining positive learning gain for the young physicians under training.

### Acknowledgments

I would like to acknowledge Dr Steven Stoltz, the director of the Division of Hospital Medicine at the Department of Medicine, UCSF/Fresno Center for Medical Education and Research, for his suggestion and support to write up this perspective to disseminate its message to other medical education programs.

Also, I would like to acknowledge Dr Ivy Darden, the director of the UCSF/Fresno internal medicine residency program in Fresno, CA, for her agreement to incorporate the idea into the resident’s didactic educational sessions.

### Conflict of Interest

The author declares that they have no conflict of interest.
